# Gossypiboma: a case report

**DOI:** 10.1186/1757-1626-1-220

**Published:** 2008-10-07

**Authors:** Ali Aminian

**Affiliations:** 1 Department of General Surgery, Tehran University of Medical Sciences, Tehran, Iran

## Abstract

Gossypiboma, an infrequent surgical complication, is a mass lesion due to a retained surgical sponge surrounded by foreign-body reaction. A 27-year-old lady presented with palpable abdominal mass five years after cesarean section. Retained foreign body was diagnosed radiologically and confirmed with operation. Retained foreign body should be in the differential diagnosis of any postoperative patient who presents with pain, infection, or palpable mass.

## Background

A surgical sponge is the most common type of retained foreign body (RFB). The condition is sometimes called gossypiboma, derived from the Latin *gossypium *(cotton) and the Swahili *boma *(place of concealment). Two usual responses lead to the detection of a retained sponge. The first type is an exudative inflammatory reaction with the formation of an abscess and usually leads to early detection and surgical removal. The second type is aseptic with a fibrotic reaction to the cotton material and development of a mass [1].

In the abdomen the sponge can be surrounded by omentum and intestines, which attempt to encapsulate it. The exerted pressure and irritation on the bowel loops can lead to necrosis of the intestinal wall and the sponge erodes partially or entirely into the lumen of the bowel. This process can lead to obstruction or fistula. Patients develop symptoms of abdominal pain, nausea, vomiting, anorexia, and weight loss resulting from obstruction or a malabsorption type syndrome caused by the multiple intestinal fistulas or intraluminal bacterial overgrowth [1,2].

## Case presentation

**Figure 1:**
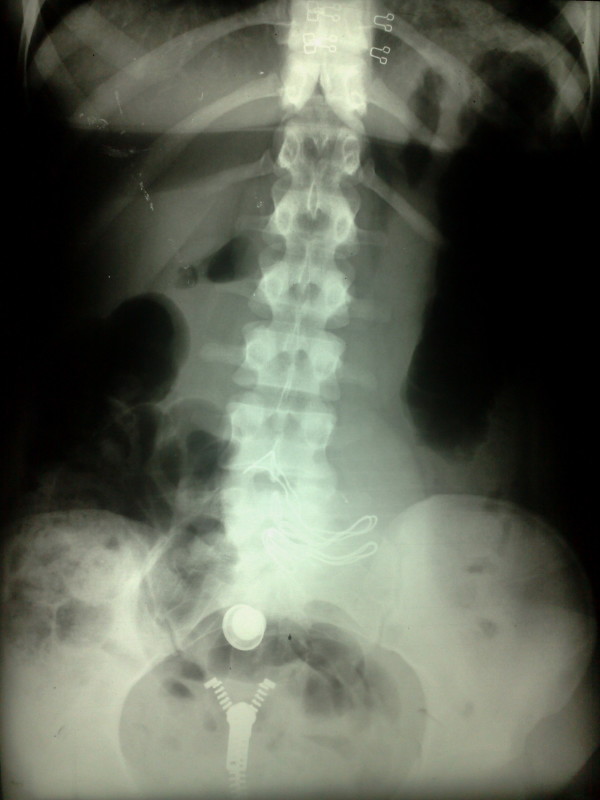
Plain X-ray of the abdomen showing the radio-opaque marker of the retained gauze in the center of abdomen.

**Figure 2:**
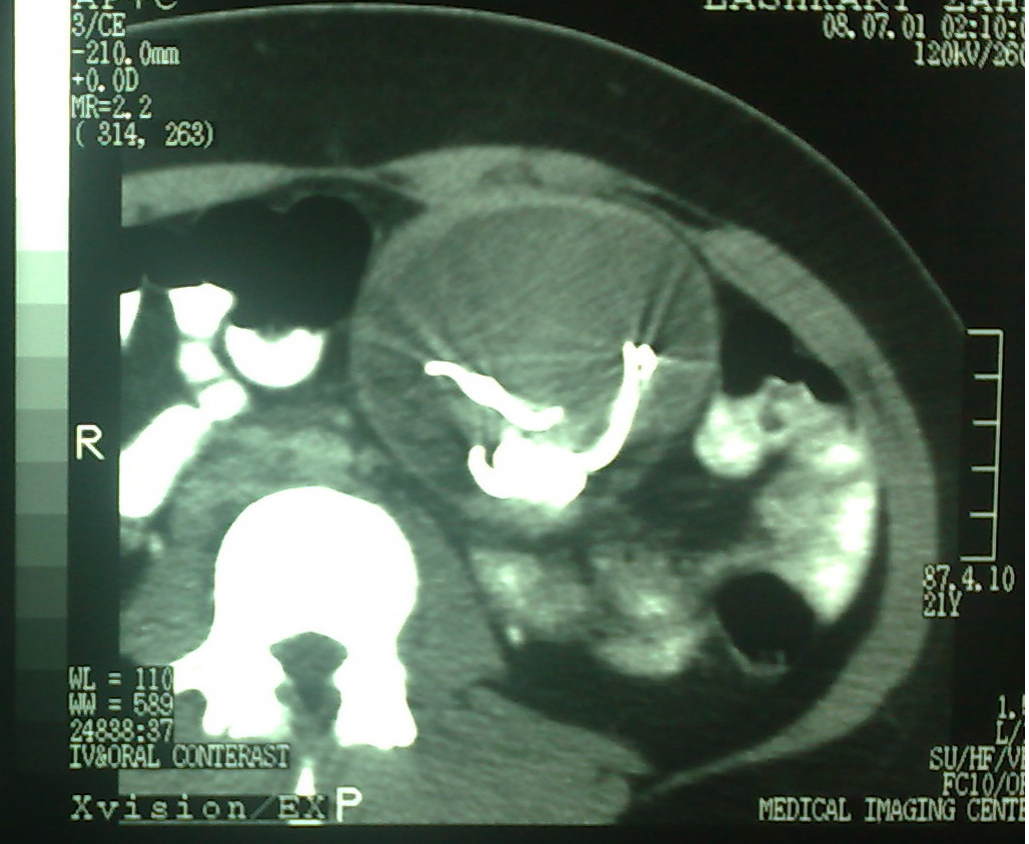
Abdominal CT scan showing a round well-defined soft-tissue mass containing an internal high-density area in the mid-abdomen.

**Figure 3:**
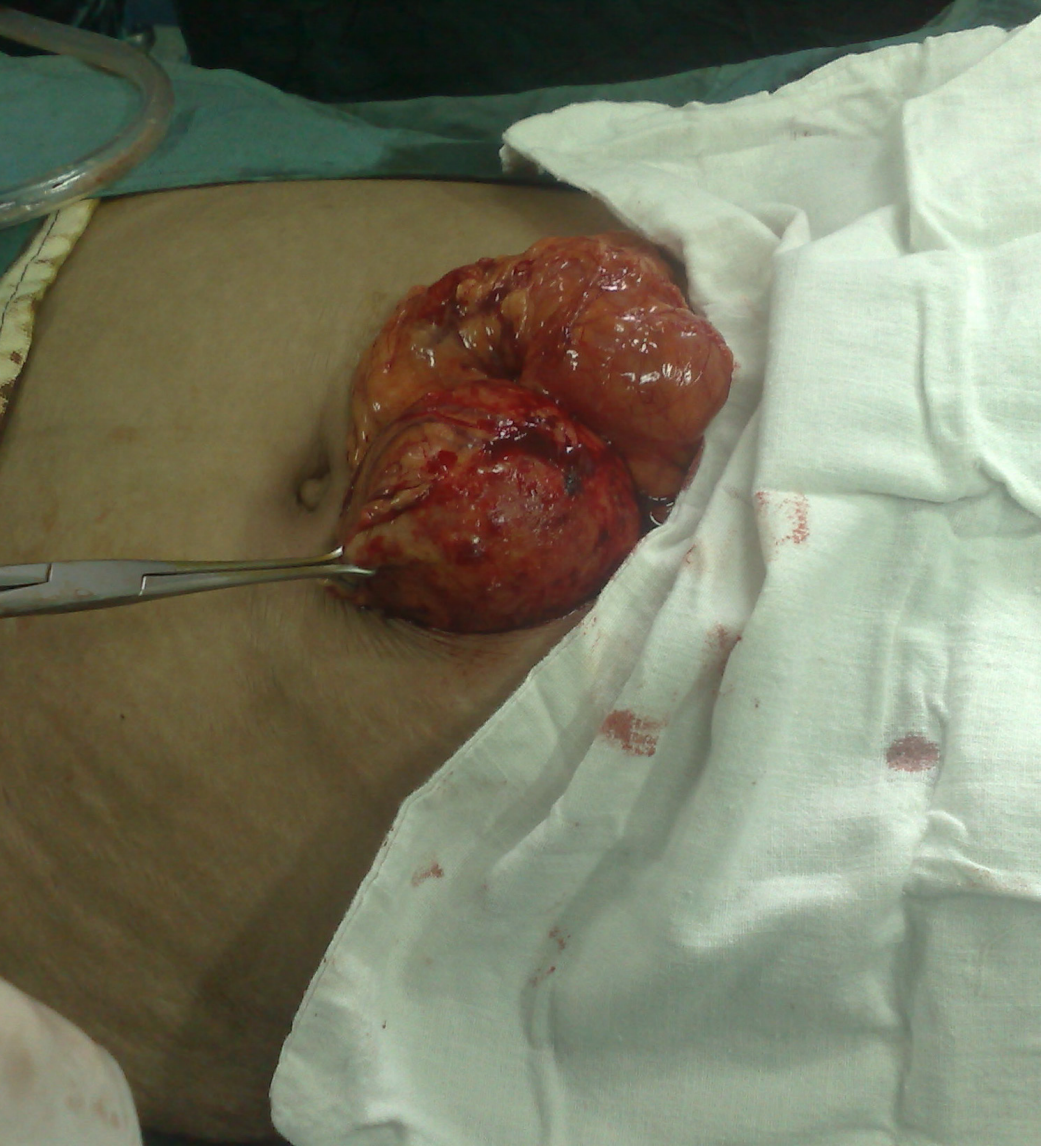
Mini-laparotomy revealed gossypiboma (grasped by the clamp).

**Figure 4:**
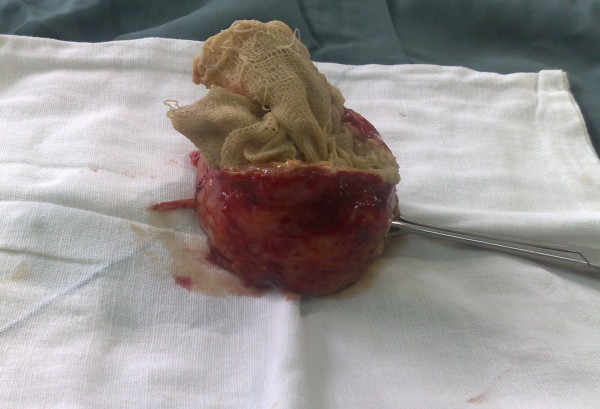
Surgical specimen (gossypiboma).

A 27-year-old lady presented with discomfort in periumbilical area since one month ago. The only positive point in her previous history was a cesarean section five years back. Vital signs were normal. On abdominal examination, a round mobile mass was palpable. All routine lab data were normal. Abdominal X-ray was in favor of retained sponge (figure 1). CT scan confirmed the diagnosis (figure 2). Exploratory laparotomy revealed an encapsulated sponge surrounded by omentum, which was removed (figure 3, 4). Postoperative course was uneventful.

## Discussion

The possibility of a RFB should be in the differential diagnosis of any postoperative patient who presents with pain, infection, or palpable mass. The first diagnostic modality to rule out a RFB should be a CT scan and often it will be the only test needed. The CT findings of a sponge usually describe a rounded mass with a dense central part and an enhancing wall. Other features of retained sponges or towels include a whorl-like appearance with trapped air bubbles and cystic masses with infolded densities. MRI features can be confusing because the radiopaque marker is not magnetic or paramagnetic so is not visible [1].

Clinicians usually think that the diagnosis of a RFB on an intraoperative radiograph is easy and obvious, but often this is not the case. Intraoperative radiographs can be of poor quality, especially in obese patients. Correctly identifying a sponge on a radiograph can be difficult. The surgical markers may become twisted or folded and present an unusual image [3]. For instance, in a report of 13 patients with a retained sponge, the radiopaque marker inside the sponge was seen in only 9 radiographs and even then was not immediately recognized for what it was [4]. Markers have been misinterpreted as calcifications, intestinal contrast material, wires, or surgical clips [1].

The usual treatment of a RFB is removal. Reopening the previous operative site is one possibility, but endoscopic or laparoscopic approaches may be attempted [5].

One possible complication during surgical removal of RFB is perforation of adherent bowels, which may be missed. We had another case with retained two surgical towels during emergency cesarean section. Her surgeon removed the towels through a small incision. However, she was admitted in our service three days later with clinical picture of generalized peritonitis. Explorative laparotomy revealed a missed small bowel perforation.

In some instances the attempt to remove the retained foreign body may cause more harm than the item itself, although in these circumstances the foreign body is usually a needle or small part of a surgical item. In these cases, removal is not recommended. Rarely is this an appropriate course of action for a retained sponge, which should always be removed [1].

Recently, *New England Journal of Medicine *published an article about risk factors of RFBs. Of the 8 risk factors the authors identified (emergency operation, unexpected change in operation, more than one surgical team involved, change in nursing staff during procedure, body mass index (BMI), volume of blood loss, female sex, and surgical counts) only 3 were found to be statistically significant by multivariate logistic regression. The 3 significant risk factors were emergency surgery, unplanned change in the operation, and BMI. The counting of sponges and instruments was not a significant predictor in the multivariate model. Although all 3 factors were significant, the 9-fold increase in risk associated with emergency surgery was impressive. In addition, in 88% of the cases where there was a RFB and counts were performed, the counts were falsely called correct. The authors recommended "radiographic screening" at the end of high risk cases as a possible adjunct to improve detection of RFB [6]. Surgeons should place radiologically detectable sponges and towels in the surgical site, carefully consider the use of small sponges in large cavities, and perform a methodical wound examination each and every time before they begin to close the wound [1].

New technologies are being developed that will hopefully decrease the incidence of RFB. An electronic article surveillance system has been examined which uses a tagged surgical sponge that can be identified electronically [7]. Bar codes can be applied to all sponges, and with the use of a bar code scanner the sponges can be counted on the back table. The use of radiofrequency identification systems holds much hope for application in the area of detection of sponges [1].

## Conclusion

RFB should be considered in the differential diagnosis of any postoperative patient who presents with pain, infection, or palpable mass. Identifying a sponge on an intraoperative radiograph is difficult. The best diagnostic modality to rule out a RFB should be a CT scan. One possible complication during surgical removal of RFB is missed perforation of adherent bowels.

## Abbreviations

RFB: Retained foreign body; BMI: Body mass index.

## Consent

Written informed consent was obtained from the patient for publication of this case report and accompanying images. A copy of the written consent is available for review by the Editor-in-Chief of this journal.

## Competing interests

The author declares that they have no competing interests.

## Authors' contributions

AA managed the patient and prepared the manuscript.
